# Neutrophil–lymphocyte ratio for the prediction of histological chorioamnionitis in cases of preterm premature rupture of membranes: a case-control study

**DOI:** 10.1186/s12884-021-04101-z

**Published:** 2021-09-27

**Authors:** Greta Balciuniene, Greta Kvederaite-Budre, Violeta Gulbiniene, Irena Dumalakiene, Rita Viliene, Ingrida Pilypiene, Grazina S. Drasutiene, Diana Ramasauskaite

**Affiliations:** 1 Clinic of Obstetrics, and Gynecology, Institute of Clinical Medicine, Faculty of Medicine of Vilnius University, M.K. Ciurlionio st. 21, 03101 Vilnius, Lithuania; 2 Center of Obstetrics and Gynecology, Vilnius University Hospital Santaros Klinikos, Santariskiu st. 2, 08661 Vilnius, Lithuania; 3 Department of Immunology, State Research Institute Centre for Innovative Medicine, Santariskiu st. 5, 08410 Vilnius, Lithuania

**Keywords:** chorioamnionitis, preterm premature rupture of membranes, neutrophil-lymphocyte ratio, white blood cell, C-reactive protein

## Abstract

**Background:**

The neutrophil-lymphocyte ratio (NLR) is easily calculated blood test parameter, which can be used as marker to predict many inflammatory disorders. The aim of this study was to assess and compare the NLR in maternal blood with the white blood cell (WBC) count and C-reactive protein (CRP) concentration for the prediction of histological chorioamnionitis.

**Methods:**

This was a case-control study of 137 woman with preterm premature rupture of membranes (PPROM) at a gestational age between 22^+ 0^ and 34^+ 6^ weeks. Blood samples, collected less than 48 h before delivery and at least 48 h after the administration of corticosteroids, were selected for the analysis. The NLR was calculated by dividing the number of neutrophils by the number of lymphocytes. Chorioamnionitis was diagnosed by the histopathological evaluation of placental membranes and chorionic plate.

**Results:**

Patients with diagnosed histological chorioamnionitis (HCA) had significantly higher levels of WBC, CRP and NLR (p-value < 0.001). Levels of WBC, CRP and NLR predicted HCA with an area under the curve (AUC) of 0.81, 0.81 and 0.89, respectively. NLR had statistically significantly higher AUC than WBC, but no significant difference was found between AUCs of NLR and CRP. The cut-off level of NLR was found to be 5,97, which had a sensitivity of 77 % and a specificity of 95 %.

**Conclusion:**

NLR has a good predictive value for HCA and could be used as an additional diagnostic marker for predicting histological chorioamnionitis in cases with preterm premature rupture of membranes before 34 weeks of gestation.

## Background

Preterm premature rupture of the membranes (PPROM) is the spontaneous rupture of foetal membranes during pregnancy prior to 37 weeks of gestation. One of the most serious complications of PPROM is chorioamnionitis, which complicates almost half of all PPROM cases [1] and is associated with increased risks of neonatal morbidity and mortality [2, 3]. Induction of labour is the most effective intervention for protecting the foetus against intrauterine infection, but preterm delivery, particularly before 32 weeks of gestation, is associated with high risks of neonatal morbidity and mortality because of foetal immaturity [3, 4]. Thus, when choosing the method of PPROM management, the benefits of pregnancy prolongation and the risk of chorioamnionitis must be balanced.

The white blood cells (WBC) and C-reactive protein (CRP) are inflammatory markers used worldwide for the early diagnosis of chorioamnionitis. A systemic review and meta-analysis performed by Sabogal with colleagues demonstrated the low sensitivity (51 %) and specificity (65 %) of leucocytosis for the diagnosis of histologic chorioamnionitis (HCA) [5]. Another systemic review by Trochez-Martinez and colleagues showed controversial results for the prediction value of CRP for chorioamnionitis [6]. Other serum inflammatory markers, such as soluble intercellular adhesion molecule-1, interleukin-6, matrix metalloproteinase-9, tissue inhibitor of metalloproteinases-1, angiopoietin-2, and insulin like growth factor binding protein-2 have also been found to be associated with chorioamnionitis, but these laboratory tests are not performed routinely [7–9].

The neutrophil–lymphocyte ratio (NLR) is a marker of systemic inflammation that is calculated by dividing the absolute neutrophilic count by the absolute lymphocyte count [10]. The NLR may be used to diagnose various diseases, such as congenital infections and COVID-19 infection and as the diagnostic and prognostic tool for sepsis [10–12]. It is an inexpensive, universal biomarker derived from a routinely performed complete blood count.

The aim of this study was to assess and compare the NLR in maternal blood with the WBC and CRP concentration to determine the best maternal blood inflammatory marker for predicting HCA.

## Materials and methods

This case–control study included 185 patients who were diagnosed with PPROM prior to 34 weeks of gestation and were admitted to Vilnius University Hospital Santaros Klinikos between July 2017 and July 2019. The study was approved by the Vilnius Regional Biomedical Research Ethics Committee (2017-07-04 No. 158200-17-931-434), and all participants signed an informed consent form before enrolment.

The inclusion criteria were as follows: (1) maternal age ≥ 18 years; (2) singleton gestation; (3) gestational age 22^+ 0^–33^+ 6^ weeks; (4) diagnosed with PPROM; and (5) the absence of a maternal hypertensive disorder, gestation diabetes and intrahepatic cholestasis. Exclusion criteria included (1) multiple gestations; and (2) foetal malformations.

Clinical characteristics such as maternal age, gravidity, parity, gestational age, newborn birthweight, Apgar score, and umbilical cord arterial pH were retrieved from the patients’ medical records.

**Fig. 1:**
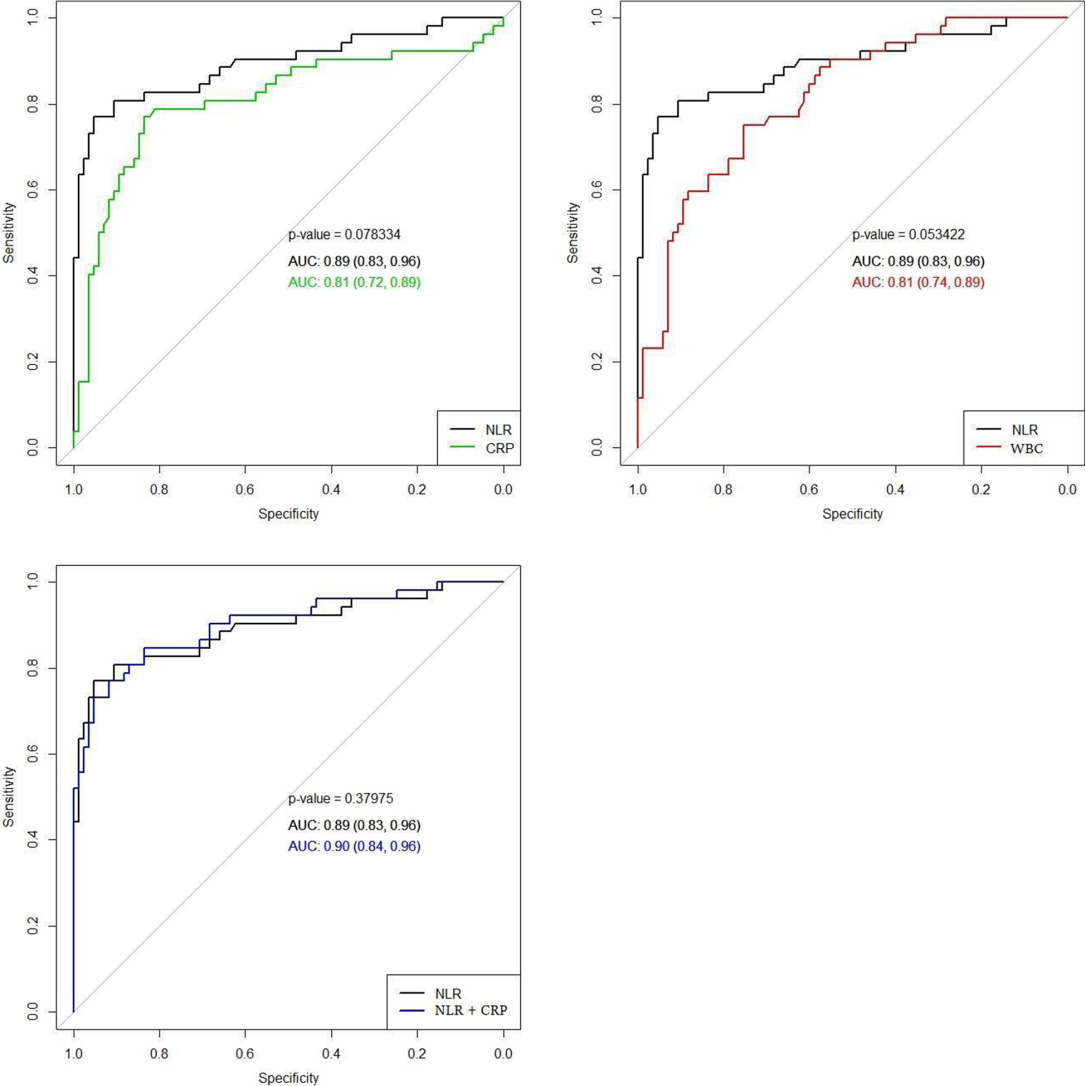
Comparison of receiver operating characteristic (ROC) curves of blood inflammatory markers. NLR, neutrophil–lymphocyte ratio; CRP, C-reactive protein; WBC, white blood cells; AUC, area under the curve

According to the institution’s standard protocol, all patients with diagnosed PPROM prior to 34 weeks of gestation were managed with antibiotics, a single course of antenatal corticosteroids, and tocolytics. Intravenous ampicillin 2 g and oral erythromycin 250 mg every 6 h were used for 48 h. The patients were then placed on oral amoxicillin 500 mg every 8 h and erythromycin 250 mg every 6 h to complete a 7-day course of antibiotic therapy. Two doses of 12 mg of dexamethasone given intramuscularly 12 h apart were used for accelerating fetal lung maturation. We analysed blood samples collected 24–48 h before delivery. Additionally, due to the presence of corticosteroid-induced leucocytosis, we only analysed blood samples that were collected at least 48 h after the administration of corticosteroids. Thus, 137 participants were included in the final analysis (Fig.1).

According to the institution’s standard protocol, all postpartum placentas were examined histologically after preterm delivery. Classification of placental lesions was based on Amsterdam Placental Workshop Group criteria [13], grading and staging of the inflammation was performed according to the diagnostic criteria proposed by the Perinatal Section of the Society for Pediatric Pathology [14].The occurrence of histological chorioamnionitis was determined based on the presence of maternal neutrophil infiltration in the amnion, chorion, and parietal decidua. Based on the histological analysis, patients were grouped into the histological chorioamnionitis group (Group I), when acute placental infectious inflammatory lesions were found, or the control group without these histological changes (Group II).

Data analysis was performed using R package (version 4.0.3) (R Core Team, 2020). The distribution of the data was determined by the Shapiro–Wilk test. All continuous variables were not normally distributed and are presented as the medians and interquartile ranges (IQRs). The categorical data are expressed as frequencies and percentages. Differences were compared between the two groups using the Mann–Whitney U test for continuous variables and the Pearson Chi-square test for categorical variables. Receiver operating characteristic (ROC) curves were constructed to estimate the ability of variables to differentiate between the groups. The area under the curve (AUC) was calculated to indicate the average sensitivity of a marker over the entire ROC curve. The DeLong test was used to compare the AUCs of the different models. The best cut-off values to predict the outcome were determined by the Youden index. A multivariate logistic regression analysis was performed to evaluate independent prognostic factors associated with HCA. *p*-Values of < 0.05 were considered statistically significant for all tests.

## Results

The study included 137 patients with preterm premature rupture of the membranes prior to 34 weeks of gestation: 52 patients in Group I and 85 patients in Group II. The clinical characteristics of the patients and their distribution within the groups are listed in Table 1. Maternal age, gravidity, parity, gestational age, neonatal birthweight, and umbilical cord arterial pH were similar between groups and did not differ statistically. Newborns in Group I, where histological chorioamnionitis was diagnosed, had lower Apgar scores (*p*-value = 0.01). Also, the time from steroid administration to delivery was longer in Group I than in Group II (*p*-value = 0.008).

**Table 1:** Characteristics of Patients According to the Groups. PPROM, preterm premature rupture of the membranes; Group I, patients with diagnosed histological chorioamnionitis; Group II, patients without diagnosed histological chorioamnionitis

Characteristics	Group I (n = 52)	Group II (n = 85)	*p*-value
Age of mother (years)	31 (28–34)	31 (27–35)	0.89
Primigravida, n (%)	18 (34.6)	36 (42.4)	0.34
Multigravida, n (%)	34 (65.4)	49 (57.6)	0.34
Primiparous, n (%)	23 (44.2)	46 (54.1)	0.26
Multiparous, n (%)	29 (55.8)	39 (45.9)	0.26
Gestational age at birth (weeks)	32 (27–34)	33 (31–34)	0.06
Birthweight (g)	1770 (1170–2203)	1925 (1510–2310)	0.12
Apgar score < 7 at 5 min, n (%)	6 (11.5)	1 (1.2)	0.01
Umbilical cord arterial pH	7.35 (7.29–7.4)	7.32 (7.26–7.37)	0.08
Clinical chorioamnionitis, n (%)	4 (7.7)	1 (1.2)	0.05
Latency between PPROM and delivery (hours)	148.3 (91.1-246.5)	93.5 (65.1-166.05)	0.007
Time from steroid administration to delivery (hours)	134.5 (78.05–233)	89 (52.01–152)	0.008

The white blood cell (WBC), neutrophil and lymphocyte counts, C-reactive protein (CRP) concentration, and the NLR are shown in Table 2. The WBC and neutrophil counts, and CRP concentration were statistically significantly higher in Group I than in Group II. The NLR was also higher in Group I, whereas the level of lymphocytes was lower in Group I than in Group II.

**Table 2:** The levels of blood inflammatory markers. Data are presented as medians (interquartile ranges). Group I, patients with diagnosed histological chorioamnionitis; Group II, patients without diagnosed histological chorioamnionitis

Blood inflammatory marker	Group I (n = 52)	Group II (n = 85)	*p*-value
White blood cells (cells x 10^9^/L)	14.58 (12.56–16.6)	10.68 (8.78–12.57)	< 0.001
Neutrophils (cells x 10^9^/L)	12 (9.75–13.9)	7.5 (6.1–9.44)	< 0.001
Lymphocytes (cells x 10^9^/L)	1.5 (1.13–1.9)	2 (1.6–2.4)	< 0.001
C-reactive protein (mg/L)	15.5 (8.71–38.38)	3.66 (2.23–6.73)	< 0.001
Neutrophil–lymphocyte ratio	8.01 (6.18–9.72)	4.09 (3.3–4.93)	< 0.001

To assess the relative importance of blood inflammatory markers in the prediction of HCA, we performed a multiple logistic regression analysis. CRP and NLR were found to be the independent variables and were significantly associated with the occurrence of HCA (Table 3).

**Table 3:** Adjusted risk factors for the occurrence of histological chorioamnionitis in patients with preterm rupture of the membranes prior to 34 weeks of gestation. CI, confidence interval

Blood inflammatory markers	Adjusted odds ratio (95 % CI)	*p*-value
White blood cells	1.84 (0.35–9.7)	0.47
Neutrophils	1.69 (0.06–2.77)	0.35
Lymphocytes	0.21 (0.41–61.49)	0.21
C-reactive protein	1.07 (1.03–1.1)	< 0.001
Neutrophil-lymphocyte ratio	2.59 (1.85–3.62)	< 0.001

ROC curves were constructed to determine the ability of CRP, WBC and NLR to differentiate between groups. Figure 1 shows a comparison of the ROC curves for the prediction of histological chorioamnionitis. CRP and NLR predicted the occurrence of histological chorioamnionitis with AUCs of 0.81 and 0.89, respectively. The DeLong test demonstrated no statistically significant difference between the ROC curves of CRP and NLR (*p*-value = 0.08). Additionally, we constructed a ROC curve of the WBC and compared it with the ROC curve of the NLR. The results were approaching an acceptable significance level (*p*-value = 0.053). Moreover, we assessed the effectiveness of CRP as an additional diagnostic marker to NLR for predicting the occurrence of HCA. This model predicted the occurrence of HCA with an AUC of 0.9 but did not differ significantly from the AUC of NLR alone (*p*-value = 0.38).

The optimum cut-off values for CRP, WBC and NLR were identified using the Youden index. Regarding the prediction of histological chorioamnionitis, the cut-off value was found to be 8.5 mg/L for CRP, 12.62 × 10^9^/L for WBC and 5.97 for NLR. The sensitivity, specificity, positive predictive value (PPV), and negative predictive value (NPV) of the markers are demonstrated in Table 4.

**Table 4:** Diagnostic indices of C-reactive protein, white blood cell count, and the neutrophil–lymphocyte ratio in study subjects. CI, confidence interval; PPV, positive predictive value; NPV, negative predictive value

Blood inflammatory markers	Cut-off value	Sensitivity % (95 % CI)	Specificity % (95 % CI)	PPV % (95 % CI)	NPV % (95 % CI)
C-reactive protein	8.5	84 (74–91)	77 (63–87)	74 (60–85)	86 (76–92)
White blood cells	12.62 × 10^9^	75 (65–84)	75 (61–86)	65 (65–77)	83 (73–91)
Neutrophil–lymphocyte ratio	5.97	77 (63–97)	95 (88–99)	91 (78–97)	87 (79–93)

## Discussion

The NLR has been proposed as an additional infection marker and a potential parameter for predicting bacterial infection. The NLR increases following the progression of inflammatory disease: chemokines release neutrophils from the bone marrow and increase their life span in the blood [15, 16], while increased levels of adrenocorticotropic hormone, cortisol, catecholamines, and corticosteroids reduce the lymphocyte count [17].

Our study demonstrates that an increased NLR is an appropriate indicator for the prenatal diagnosis of HCA. According to the ROC curve analysis, the prognostic value of NLR does not differ significantly from that of the CRP, but it has greater prognostic value than the WBC and may be used as additional marker to predict HCA. The optimal NLR cut-off value to predict HCA was found to be 5.97 with a sensitivity of 77 % and a specificity of 95 %.

To our knowledge, other published studies on the use of the NLR in the prediction of chorioamnionitis have been sporadic [18, 19]. Kim and colleagues evaluated the predictive value of the NLR in the placental inflammatory response and found that the NLR had a better diagnostic value than the maternal serum CRP or WBC counts. The optimal NLR cut-off value to predict the placental inflammatory response was found to be 6.48, similar to our result, but that study included all patients who underwent a preterm delivery between 24 and 37 weeks of gestation. Additionally, those authors showed that patients with a high NLR were at risk of impending preterm delivery in the context of a normal CRP level, and NLR together with CRP can help to predict poor pregnancy outcomes in patients with a placental inflammatory response [18].

Recent studies focused on the ability of the NLR to predict preterm delivery. Kim and colleagues found that a combined model consisting of cervical length and NLR had a higher predictive value for spontaneous preterm delivery than using cervical length alone or other inflammatory markers such as CRP and WBC [20]. A study by Akgun et al. demonstrated that an elevated NLR is associated with preterm delivery and lower birthweight. The authors hypothesized that an elevated NLR would be affected by the maternal hyperinflammatory state that led to foetal growth restriction and early initiation of delivery [21].

Patients with an imminent preterm delivery prior to 34 weeks of gestation receive corticosteroid therapy to accelerate foetal lung development. Corticosteroids are known to increase the WBC and predominantly the neutrophil count. The biological effects that contribute to the increase in neutrophils are the release of immature neutrophils from the bone marrow into the circulation, the demargination of neutrophils from the endovascular lining, the delayed migration of neutrophils into tissue, and the lower rate of apoptosis [22–24]. Leucocytosis is induced for up to 24–48 h after corticosteroid administration [25]. Thus, the NLR is a misleading predictive factor of infection during this period.

The NLR changes during some pregnancy-related disorders. The NLR may increase in preeclampsia, gestational diabetes mellitus, and intrahepatic cholestasis [26–28]. Furthermore, Orgul and colleagues evaluated how the NLR changes in pregnant women who are administrated magnesium sulfate for foetal neuroprotection and found that the NLR increased 6 h after starting magnesium sulfate [29]. Thus, the NLR should be evaluated carefully in the presence of pregnancy-related disorders, such as preeclampsia, gestational diabetes mellitus, and intrahepatic cholestasis and in cases where magnesium sulfate treatment occurs.

### Practical recommendation

Our clinical expectation is that the NLR together with other maternal blood inflammatory markers will improve the prediction of histological chorioamnionitis. NLR analysis is simple, inexpensive, and easily obtained.

### Strengths and limitations

To the best of our knowledge, this is the first study to evaluate the NLR for the prediction of HCA in patients with PPROM prior to 34 weeks of gestation. Additionally, this study excluded blood samples affected by the administration of corticosteroids and cases with preeclampsia, gestational diabetes mellitus, and intrahepatic cholestasis. The limitation of our study is its retrospective design. Further prospective cohort studies are required to evaluate our result and to produce stronger evidence. Moreover, chorioamnionitis was defined by histological examination of the placenta. Although histological chorioamnionitis is the gold standard for diagnosing intrauterine infections [30], there is controversy as to whether histological chorioamnionitis is correlated with higher rates of neonatal morbidity and mortality [30–32].

## Conclusions

The NLR has a good predictive value for the occurrence of HCA and could be used as an additional diagnostic marker for predicting histological chorioamnionitis in cases with preterm premature rupture of the membranes prior to 34 weeks of gestation.

## Data Availability

The data that support the findings of this study are available on request from corresponding author. The data are not publicly available due to privacy or ethical restrictions.

## Abbreviations

NLR: neutrophil-lymphocyte ratio
WBC: white blood cell
CRP: C-reactive protein
HCA: histological chorioamnionitis
PPROM: preterm premature rupture of the membranes
IQR: interquartile range
ROC: receiver operating curve
AUC: area under the curve
